# Supplementary material for: Population morphology implies a common developmental blueprint for Drosophila motion detectors
Source: PLoS Comput Biol. 2026 Aug 31;22(8):e1014657. doi: 10.1371/journal.pcbi.1014657 (PMC13557502; doi:10.1371/journal.pcbi.1014657)
Supplement: Supplementary file 1 [file pcbi.1014657.s001.pdf]

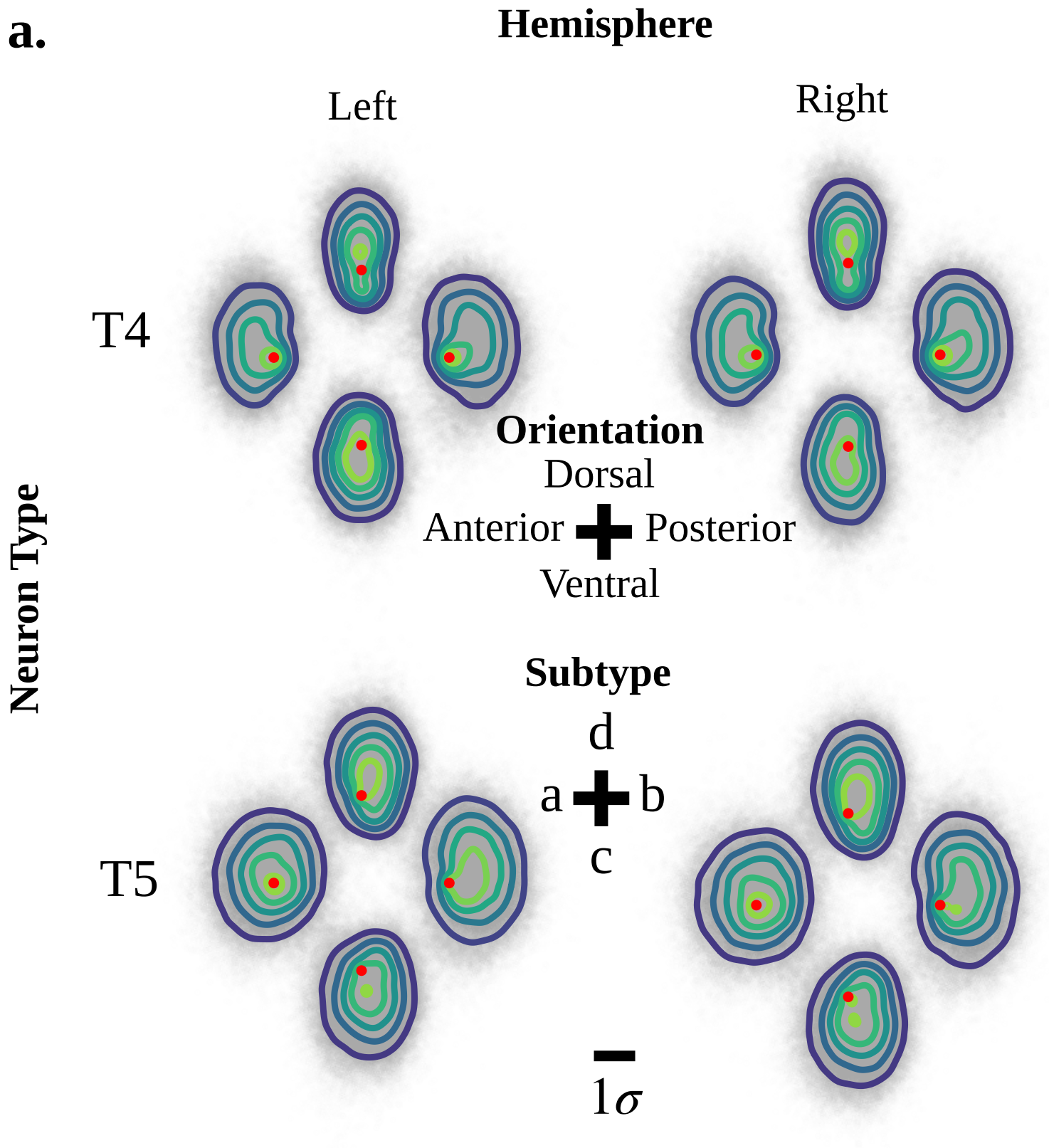
a.
Hemisphere
Left
Right
T4
Orientation
Dorsal
Anterior
Posterior
Ventral
Neuron Type
Subtype
d
a
b
c
T5
1σ
