# Supplementary material for: Population morphology implies a common developmental blueprint for Drosophila motion detectors
Source: PLoS Comput Biol. 2026 Aug 31;22(8):e1014657. doi: 10.1371/journal.pcbi.1014657 (PMC13557502; doi:10.1371/journal.pcbi.1014657)
Supplement: Supplementary file 2 [file pcbi.1014657.s002.pdf]

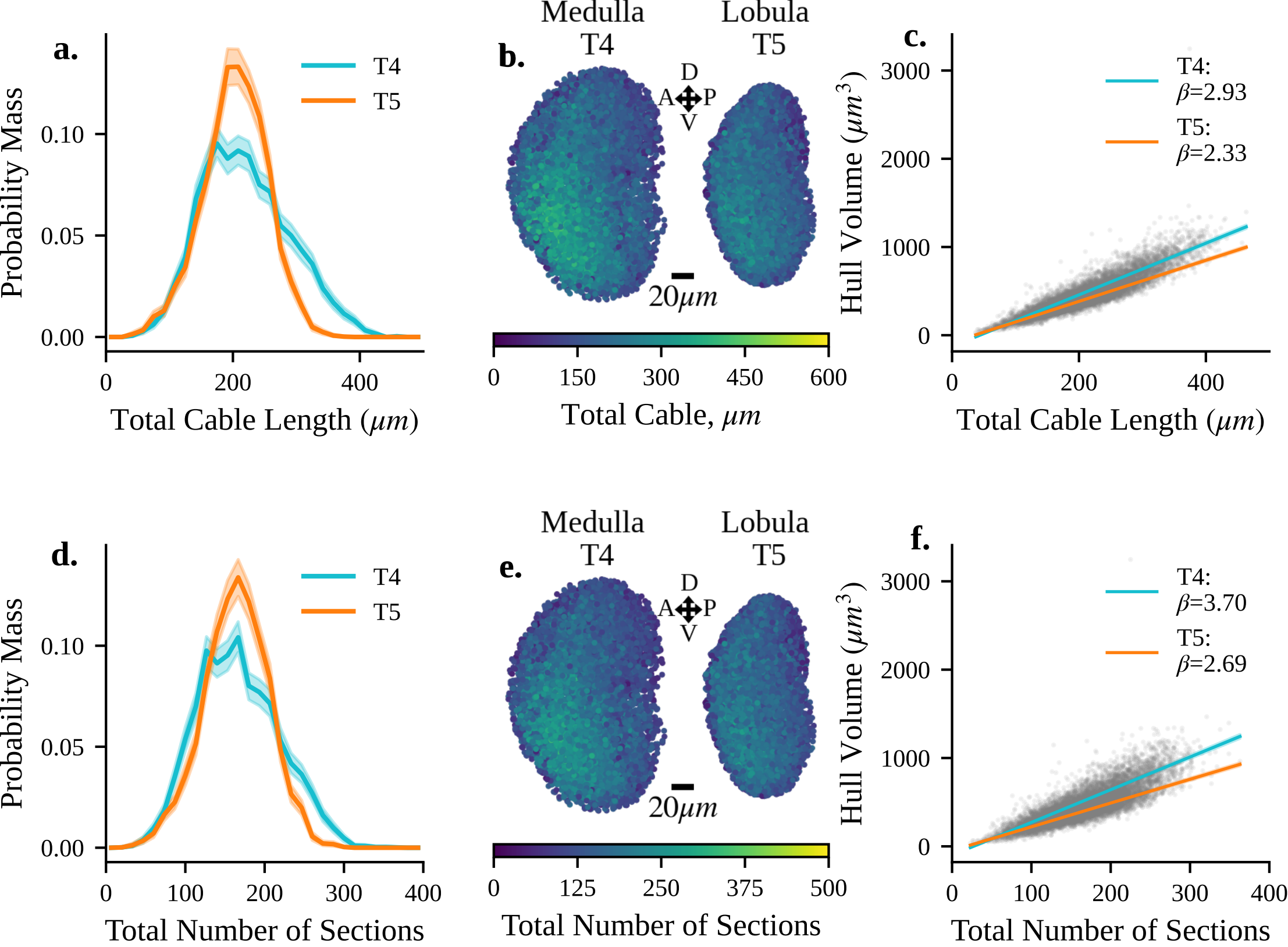

a.
Probability Mass
0.00
0.05
0.10
0
200
400
Total Cable Length (μm)
T4
T5
b.
Medulla
T4
Lobula
T5
D
A
P
V
20μm
0
150
300
450
600
Total Cable, μm
c.
Hull Volume (μm^3)
0
1000
2000
3000
0
200
400
Total Cable Length (μm)
T4:
β=2.93
T5:
β=2.33
d.
Probability Mass
0.00
0.05
0.10
0
100
200
300
400
Total Number of Sections
T4
T5
e.
Medulla
T4
Lobula
T5
D
A
P
V
20μm
0
125
250
375
500
Total Number of Sections
f.
Hull Volume (μm^3)
0
1000
2000
3000
0
100
200
300
400
Total Number of Sections
T4:
β=3.70
T5:
β=2.69
