# Supplementary material for: Population morphology implies a common developmental blueprint for Drosophila motion detectors
Source: PLoS Comput Biol. 2026 Aug 31;22(8):e1014657. doi: 10.1371/journal.pcbi.1014657 (PMC13557502; doi:10.1371/journal.pcbi.1014657)
Supplement: Supplementary file 3 [file pcbi.1014657.s003.pdf]

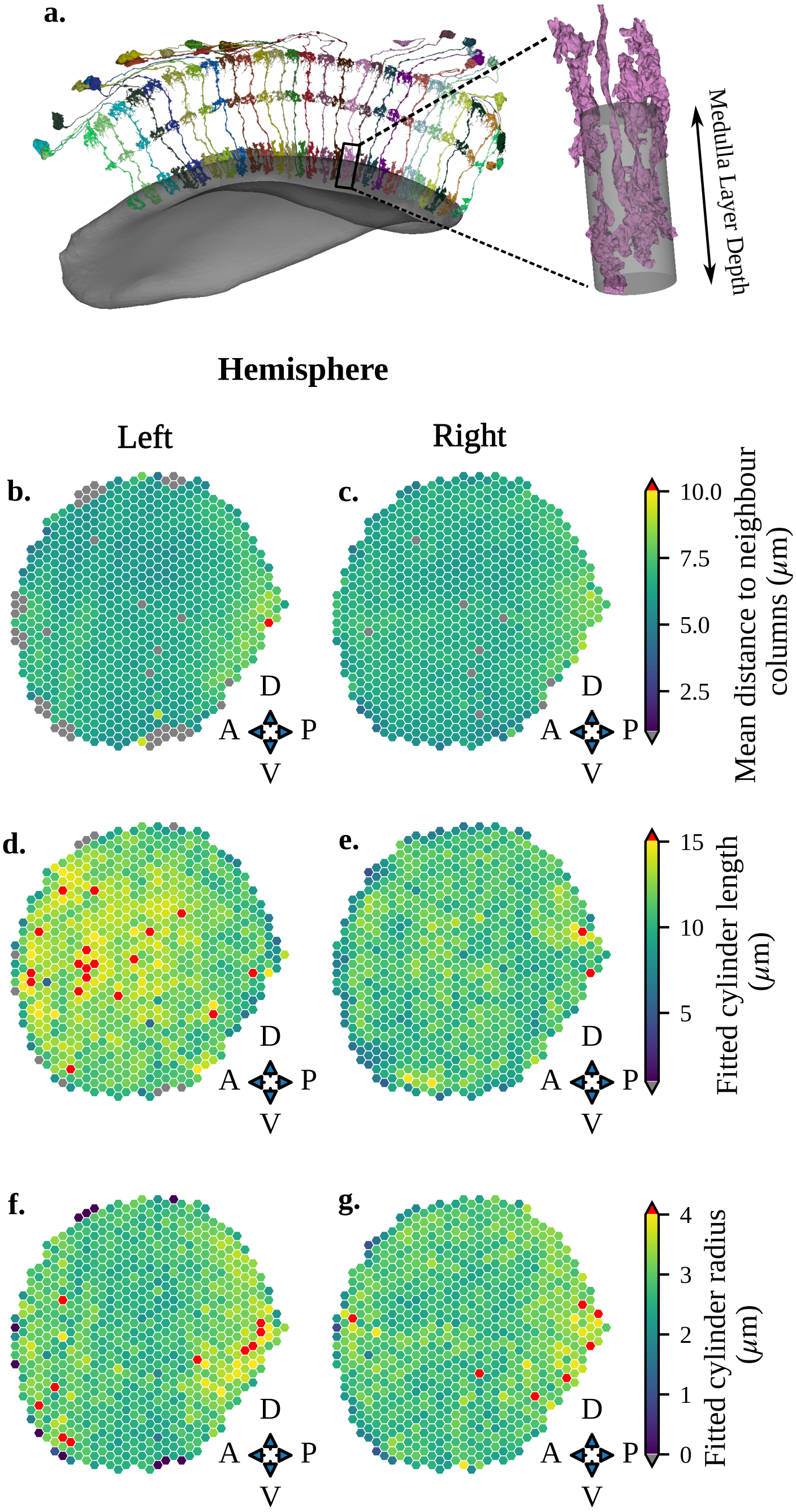
a.
Medulla Layer Depth
Hemisphere
Left
Right
b.
c.
d.
e.
f.
g.
D
A
P
V
10.0
7.5
5.0
2.5
Mean distance to neighbour columns (μm)
15
10
5
Fitted cylinder length (μm)
4
3
2
1
0
Fitted cylinder radius (μm)
