# Supplementary material for: Population morphology implies a common developmental blueprint for Drosophila motion detectors
Source: PLoS Comput Biol. 2026 Aug 31;22(8):e1014657. doi: 10.1371/journal.pcbi.1014657 (PMC13557502; doi:10.1371/journal.pcbi.1014657)
Supplement: Supplementary file 4 [file pcbi.1014657.s004.pdf]

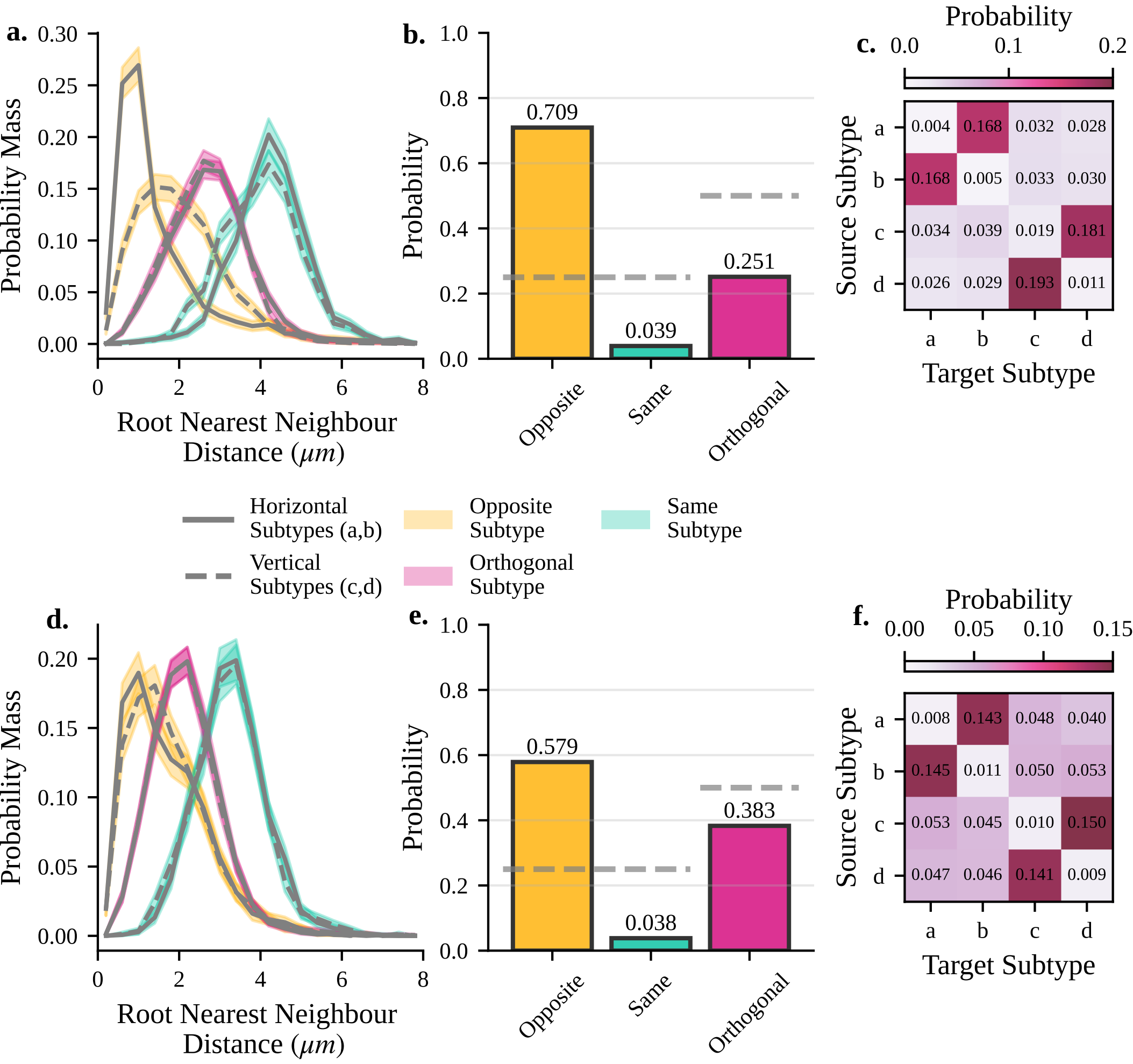
a.
Probability Mass
0.30
0.25
0.20
0.15
0.10
0.05
0.00
0
2
4
6
8
Root Nearest Neighbour Distance (μm)
b.
Probability
1.0
0.8
0.6
0.4
0.2
0.0
0.709
0.039
0.251
Opposite
Same
Orthogonal
c.
Probability
0.0
0.1
0.2
Source Subtype
Target Subtype
a
b
c
d
0.004 0.168 0.032 0.028
0.168 0.005 0.033 0.030
0.034 0.039 0.019 0.181
0.026 0.029 0.193 0.011
Horizontal Subtypes (a,b)
Vertical Subtypes (c,d)
Opposite Subtype
Orthogonal Subtype
Same Subtype
d.
Probability Mass
0.20
0.15
0.10
0.05
0.00
0
2
4
6
8
Root Nearest Neighbour Distance (μm)
e.
Probability
1.0
0.8
0.6
0.4
0.2
0.0
0.579
0.038
0.383
Opposite
Same
Orthogonal
f.
Probability
0.00
0.05
0.10
0.15
Source Subtype
Target Subtype
a
b
c
d
0.008 0.143 0.048 0.040
0.145 0.011 0.050 0.053
0.053 0.045 0.010 0.150
0.047 0.046 0.141 0.009
