# Supplementary material for: Population morphology implies a common developmental blueprint for Drosophila motion detectors
Source: PLoS Comput Biol. 2026 Aug 31;22(8):e1014657. doi: 10.1371/journal.pcbi.1014657 (PMC13557502; doi:10.1371/journal.pcbi.1014657)
Supplement: Supplementary file 5 [file pcbi.1014657.s005.pdf]

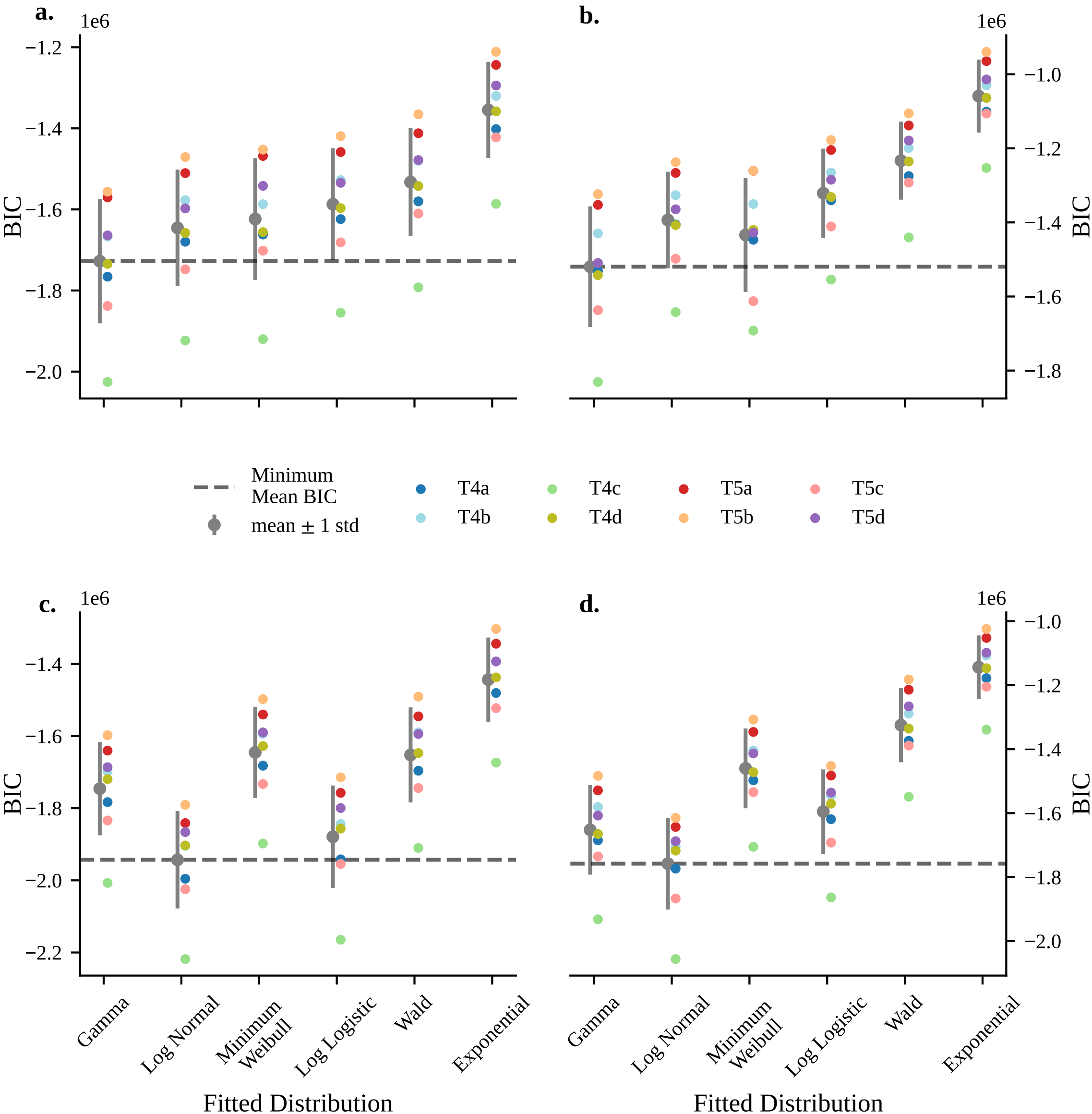

a.
b.
c.
d.
1e6
BIC
−1.2
−1.4
−1.6
−1.8
−2.0
−1.0
−2.2
Minimum Mean BIC
mean ± 1 std
T4a
T4b
T4c
T4d
T5a
T5b
T5c
T5d
Gamma
Log Normal
Minimum Weibull
Log Logistic
Wald
Exponential
Fitted Distribution
